# Prevalence of *pfhrp2* and/or *pfhrp3* Gene Deletion in *Plasmodium falciparum* Population in Eight Highly Endemic States in India

**DOI:** 10.1371/journal.pone.0157949

**Published:** 2016-08-12

**Authors:** Praveen Kumar Bharti, Himanshu Singh Chandel, Amreen Ahmad, Sri Krishna, Venkatachalam Udhayakumar, Neeru Singh

**Affiliations:** 1 National Institute for Research in tribal Health (NIRTH), Jabalpur, 482003, Madhya Pradesh, India; 2 Malaria Branch, Division of Parasitic Diseases and Malaria, Center for Global Health, Centers for Disease Control and Prevention, Atlanta, GA, 30333, United States of America; Université Pierre et Marie Curie, FRANCE

## Abstract

**Background:**

*Plasmodium falciparum* encoded histidine rich protein (HRP2) based malaria rapid diagnostic tests (RDTs) are used in India. Deletion of *pfhrp2* and *pfhrp3* genes contributes to false negative test results, and large numbers of such deletions have been reported from South America, highlighting the importance of surveillance to detect such deletions.

**Methods:**

This is the first prospective field study carried out at 16 sites located in eight endemic states of India to assess the performance of PfHRP2 based RDT kits used in the national malaria control programme. In this study, microscopically confirmed *P*. *falciparum* but RDT negative samples were assessed for presence of *pfhrp2*, *pfhrp3*, and their flanking genes using PCR.

**Results:**

Among 1521 microscopically positive *P*. *falciparum* samples screened, 50 were negative by HRP2 based RDT test. Molecular testing was carried out using these 50 RDT negative samples by assuming that 1471 RDT positive samples carried *pfhrp2* gene. It was found that 2.4% (36/1521) and 1.8% (27/1521) of samples were negative for *pfhrp2* and *pfhrp3* genes, respectively. However, the frequency of *pfhrp2* deletions varied between the sites ranging from 0–25% (2.4, 95% CI; 1.6–3.3). The frequency of both *pfhrp2* and *pfhrp3* gene deletion varied from 0–8% (1.6, 95% CI; 1.0–2.4).

**Conclusion:**

This study provides evidence for low level presence of *pfhrp2* and *pfhrp3* deleted *P*. *falciparum* parasites in different endemic regions of India, and periodic surveillance is warranted for reliable use of PfHRP2 based RDTs.

## Introduction

Malaria is a major public health problem in India, which has the highest number of malaria cases outside of Africa. Malaria prevalence in India varies between states and eight out of 35 states and union territories are contributing to 80% of total malaria cases, 85% *Plasmodium falciparum*, and 70% of deaths due to malaria in the country [1]. *P*. *falciparum* infection can become a life threatening disease, if not diagnosed early and treated [2].The introduction of malaria Rapid Diagnostic Tests (RDTs) has made it possible to obtain diagnostic results quickly and provide treatment in a timely manner. The availability of RDTs and the scale of their use in India have rapidly increased in recent years [3] along with the global increase in their use from 46 million in 2008 to 319 million in 2013 [4]. Most of the commercially available RDTs detect histidine-rich protein 2 (HRP2) which is produced during the asexual blood stage of *P*. *falciparum* but not by other species of malaria parasites making PfHRP2 based RDT species specific [5]. Some PfHRP2 based RDTs can cross react with HRP3 encoded by *pfhrp3* gene due to shared antigenic epitopes between these proteins [6]. *Pfhrp2* and *pfhrp3* are structural homologue and their respective genes are located on chromosome 8 and chromosome 13 [7]. The plasma level of PfHRP2 has been shown to be a surrogate marker for the severity of *P*. *falciparum* malaria in some studies [8] as it can indirectly reflect the parasite load of sequestered parasites [9]. It may also be used as marker to differentiate between uncomplicated and severe malaria [10].

We have evaluated various brands of RDTs in various epidemiological settings in India with differing results in the past [11, 12]. The sensitivity of various RDTs ranged from 76% to 98% [3]. One major concern with RDT performance is the presence of false negative results which can lead to misdiagnosis and failure to treat. Several factors can contribute to false negative test results such as low parasite densities [3], incorrect interpretation of RDT results, prozone effect [13], or *pfhrp2* gene deletion [14]. The first evidence for large scale *pfhrp2* and *pfhrp3* gene deletion came from Peru [15] and was further substantiated by other researchers [7, 14, 16, 17]. Such a large scale deletion of *pfhrp2* has been found only in different parts of South America. Studies conducted in Africa and Asia showed *pfhrp2* deletion in a small number of parasite isolates [18–21]. We do not know if the false negative result in various RDT evaluation studies we conducted were at least partially due to *pfhrp2* negative strains. There is very limited information about the extent of *pfhrp2* and *pfhrp3* gene deletion in *P*. *falciparum* parasites in South Asia. A small previous study conducted in Chhattisgarh state, India, revealed two out of 48 samples showed *pfhrp2* deletion [18]. It is important to monitor *pfhrp2* negative parasites present in *P*. *falciparum* populations, because in India, RDTs are used in the national malaria programme to diagnose malaria for case management in regions where microscopic diagnosis is not available. Therefore, the main objective of this study was to determine whether there is further evidence to confirm the presence of *pfhrp2 and pfhrp3* deleted *P*. *falciparum* parasites in eight highly endemic states in India.

## Material and Methods

**Fig 1:**
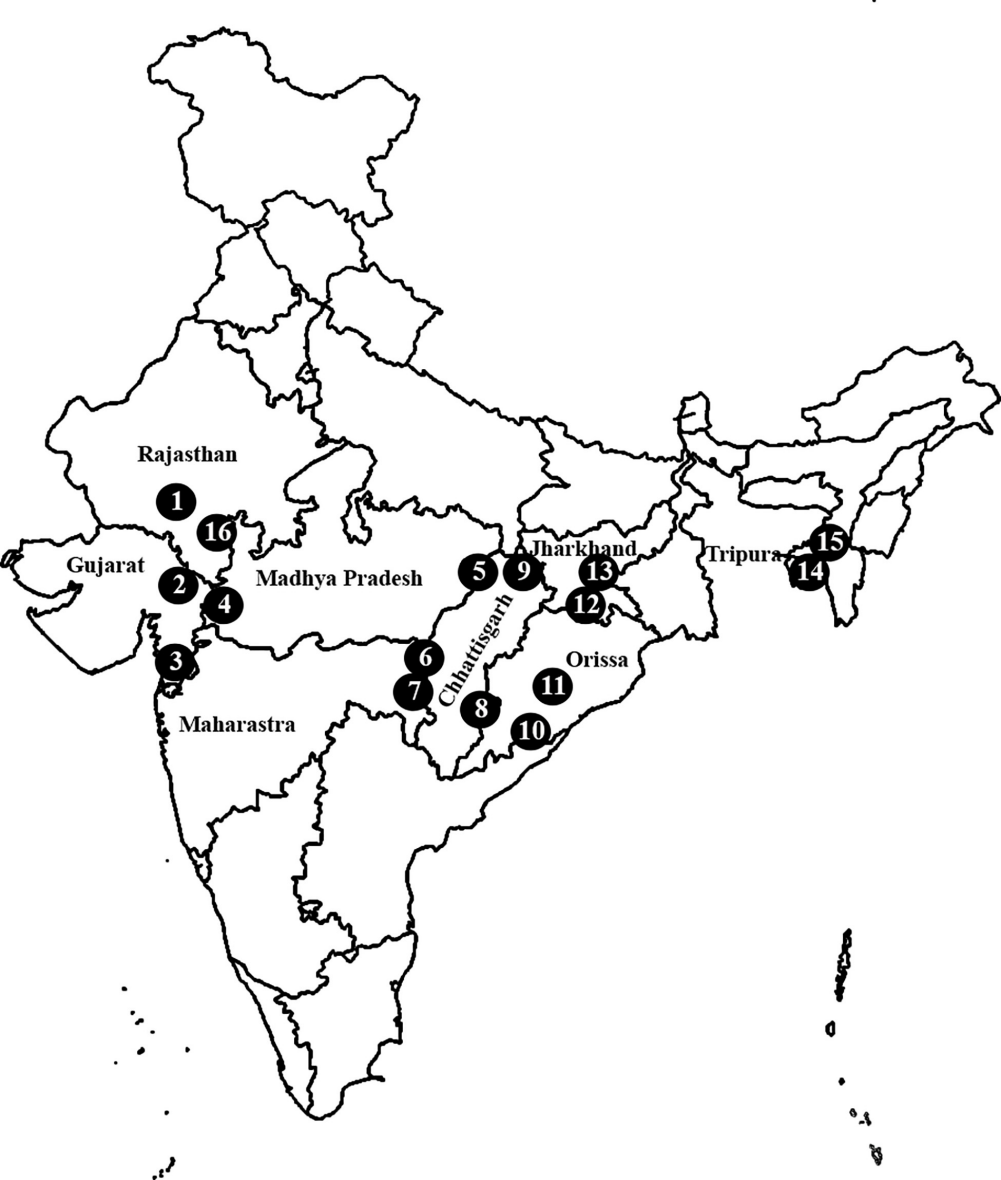
Map showing the study sites from eight malaria endemic states of India. Each state has two study sites.

We conducted a prospective study to determine the prevalence of *pfhrp2 and pfhrp3* gene deletion in *P*. *falciparum* positive samples confirmed by microscopy. Samples were collected from 16 sites in eight malaria endemic states in India (Fig 1); two sites were selected from each state one having high malaria endemicity (Annual Parasite Incident > 5) and other one having low malaria endemicity (Annual Parasite Incident <2).

Positive *P*. *falciparum* samples were collected from July to December 2014 simultaneously from all sites during the rainy season and coinciding with the main transmission season [22]. These States are Odisha (OD), Chhattisgarh (CG), Jharkhand (JH), Madhya Pradesh (MP), Maharashtra (MH), Rajasthan (RJ), Gujarat (GJ), and Tripura (TR). Two community health centres (CHCs) representing a high endemic and low endemic regions were chosen from each State. During the study, we screened all febrile patients who attended the CHC hospital requiring malaria diagnosis. The patients were enrolled under the study after obtaining written informed consent from patients or their guardians. The blood smears were stained with JSB [23] and examined under the microscope. Those found positive were treated as per existing National Vector Borne Disease Control Programme (NVBDCP) policy [22]. The inclusion criteria for sample collection included positive identification of *P*. *falciparum* mono-infection by microscopy in the blood smears of symptomatic patients over the age of 5 year. Pregnant women were excluded from the study. 1-2ml venous blood samples were collected in heparin coated vacutainer and after plasma separation samples were stored under freezing conditions for further molecular studies. The blood smears were examined independently by two microscopists and if there was a discrepancy in the result, a third expert microscopist read the slide and confirmed the results. Parasite densities were calculated according to the standard technique (Parasites/μL = no. of asexual parasites X 8000/no. of WBC counted).

To test our working hypothesis that low level of *pfhrp2* deleted parasites may be widely present in different endemic states of India, we carried out the following sample size calculation. In a previous study conducted in the Chhattisgarh State of India 4% *pfhrp2* gene deletion was reported [18]. Therefore, to estimate the prevalence of *P*. *falciparum* infected individuals carrying *pfhrp2* deleted parasites with 95% level of confidence and 25% relative precision, assuming a 4% *pfhrp2* gene deletion rate, an overall sample size of 1536 *P*. *falciparum* cases were required.

The prevalence of *pfhrp2*, *pfhrp3*, and flanking gene deletion was determined by dividing the number of isolates by the total number of enrolled *P*. *falciparum* subjects. The study was approved by ethics committee of National Institute for Research in Tribal Health (ICMR). Written informed consent was signed by participants and their parents/guardians if they were minors.

### Rapid diagnostic test

Bivalent malaria RDT was performed immediately from collected blood samples as per manufacturer’s protocol by taking 5 μL of whole blood with the help of circular loop provided by the manufacturer {SD Bioline Malaria Antigen P.f./P.v. (05FK80I-40), Bio Standard Diagnostics Pvt. Ltd., India}. The results were interpreted within the specified reading time of the manufacture’s protocol (15–30 minutes). RDT was also repeated for samples which gave negative results.

### DNA extraction

Genomic DNA was extracted from 200 μL of whole blood using QIAamp DNA blood mini Kit (Qiagen, Germany) as per the manufacturer’s protocol and stored at –20°C for further molecular biology studies.

#### Species specific PCR

Presence of *P*. *falciparum* infection in all the samples was further confirmed by PCR using 18S ribosomal RNA (rRNA) gene amplification. Species specific nested PCR was performed [24] and detailed protocol was described earlier [25]. To check the quality of isolated genomic DNA, PCR amplification of two more genes, *P*. *falciparum* merozoite surface protein 1 (*pfmsp1*), and *P*. *falciparum* merozoite surface protein 2 (*pfmsp2*), was performed using specific primers [26]. In brief, for primary PCR 5μL of genomic DNA was taken as template and for nested PCR 2μL of 1:10 diluted primary PCR product was taken as template. Primer sequences and PCR cycling conditions were used as given in Table 1. PCR reaction was performed in a 25μL reaction mixture containing 10X buffer, 1mM MgCl_2_, 0.2mM each dNTP, 0.4μM each primer, and 0.2 units of Taq polymerase (Invitrogen, life technologies) with initial denaturation at 95°C for 5 min, and final extension at 72°C for 8 min. All the PCR products were analyzed in 1.2% agarose gel and image was captured under GelDoc-It2 imager.

**Table 1:** Details of primers and PCR conditions.

S.No.	Gene	Primer Sequence (5’➔3’); Forward (F) and Reverse (R)	PCR Product length	PCR Programme						No of Cycle
				Denaturation		Annealing		Elongation		
				Temp	Time	Temp	Time	Temp	Time	
1	MSP1 (Primary)	F: CACAATGTGTAACACATGAAAG	646 bp	94°C	1 Min	55°C	1 Min	72°C	1 Min	35
		R: AGTACGTCTAATTCATTTGCAC								
	MSP1 (Nested)	F: TAGAAGCTTTAGAAGATGCAG	555 bp	94°C	1 Min	53°C	1 Min	72°C	1 Min	25
		R: GACAATAATCATTAGCACATAC								
2	MSP2 (Primary)	F: ATGAAGGTAATTAAAACATTGTC	760 bp	94°C	1 Min	53°C	1 Min	72°C	1 Min	35
		R: TTATTGAAGCAATATTACTAGAG								
	MSP2 (Nested)	F: AGCAACACATTCATAAACAATG	634 bp	94°C	1 Min	54°C	1 Min	72°C	1 Min	25
		R: CACAGTTTTCTTTGTTACCATC								
3	PF3D7_0831900 (MAL7P1.230) (Primary)	F: GATATCATTAGAAAACAAGAGCTTAG	405 bp	94°C	1 Min	63°C	1 Min	72°C	1 Min	35
		R: TATCCAATCCTTCCTTTGCAACACC								
	PF3D7_0831900 (MAL7P1.230) (Nested)	F: TATGAACGCAATTTAAGTGAGGCAG	356 bp	94°C	1 Min	65°C	1 Min	72°C	1 Min	25
		R: TATCCAATCCTTCCTTTGCAACACC								
4	PfHRP2-2	F: CAAAAGGACTTAATTTAAATAAGAG	814 bp	94°C	1 Min	55°C	1 Min	72°C	1 Min	35
		R: AATAAATTTAATGGCGTAGGCA								
5	PfHRP2-12 (Primary)	F: GGTTTCCTTCTCAAAAAATAAAG	307 bp	94°C	1 Min	58°C	1 Min	72°C	1 Min	35
		R: TCTACATGTGCTTGAGTTTCG								
	PfHRP2-12 (Nested)	F: GTATTATCCGCTGCCGTTTTTGCC	222 bp	94°C	1 Min	63°C	1 Min	72°C	1 Min	25
		R: CTACACAAGTTATTATTAAATGCGGAA								
6	PF3D7_0831700 (MAL7P1.228) (Primary)	F:AGACAAGCTACCAAAGATGCAGGTG	200 bp	94°C	1 Min	60°C	1 Min	72°C	1 Min	35
		R: TAAATGTGTATCTCCTGAGGTAGC								
	PF3D7_0831700 (MAL7P1.228) (Nested)	F: CCATTGCTGGTTTAAATGTTTTAAG	197 bp	94°C	1 Min	63°C	1 Min	72°C	1 Min	25
		R: TAAATGTGTATCTCCTGAGGTAGC								
7	PF3D7_1372100(MAL13P1.485) (Primary)	F: TTGAGTGCAATGATGAGTGGAG	287 bp	94°C	1 Min	60°C	1 Min	72°C	1 Min	35
		R: AAATCATTTCCTTTTACACTAGTGC								
	PF3D7_1372100(MAL13P1.485) (Nested)	F: GTTACTACATTAGTGATGCATTC	266 bp	94°C	1 Min	59°C	1 Min	72°C	1 Min	25
		R: AAATCATTTCCTTTTACACTAGTGC								
8	PfHRP3-2	F: AATGCAAAAGGACTTAATTC	719 bp	94°C	1 Min	55°C	1 Min	72°C	1 Min	35
		R: TGGTGTAAGTGATGCGTAGT								
9	PfHRP3-12 (Primary)	F: GGTTTCCTTCTCAAAAAATAAAA	311 bp	94°C	1 Min	53°C	1 Min	72°C	1 Min	25
		R: CCTGCATGTGCTTGACTTTA								
	PfHRP3-12 (Nested)	F: ATATTATCGCTGCCGTTTTTGCT	226 bp	94°C	1 Min	62°C	1 Min	72°C	1 Min	30
		R: CTAAACAAGTTATTGTTAAATTCGGAG								
10	PF3D7_1372400 (MAL13P1.475) (Primary)	F: TTCATGAGTAGATGTCCTAGGAG	260 bp	94°C	1 Min	55°C	1 Min	72°C	1 Min	35
		R: TCGTACAATTCATCATACTCACC								
	PF3D7_1372400 (MAL13P1.475) (Nested)	F: TTCATGAGTAGATGTCCTAGGAG	234 bp	94°C	1 Min	61°C	1 Min	72°C	1 Min	25
		R: GGATGTTTCGACATTTTCGTCG								

#### Detection of *pfhrp2* and *pfhrp3* genes

Samples showing amplification for all three genes (18S rRNA, *msp1*, *msp2*) were sequenced in the study. Samples which were microscopically and PCR positive for *P*. *falciparum* and negative by RDT were subjected to *pfhrp2*, *pfhrp3*, and flanking genes of *pfhrp2* and *pfhrp3* PCR amplification. PCR amplification of DNA fragments encompassing exon1, intron, exon2, and fragment encompassing exon2 of *pfhrp2* and *pfhrp3* genes was performed using specific primers for confirmation of deletion of these genes using previously described method [7, 27]. Primers and PCR cycling conditions used for amplification of these genes are given in Table 1.

#### Detection of genes flanking *pfhrp2* and *pfhrp3* by PCR

PCR was also performed for flanking genes, *pfhrp2* upstream (PF3D7_0831900), *pfhrp2* downstream (PF3D7_0831700), and *pfhrp3* upstream (PF3D7_1372100), *pfhrp3* downstream (PF3D7_1372400) using specific primers for confirmation of deletion of these genes [7]. PCR cycling conditions used for amplification of these genes are given in Table 1. The composition of reaction mixture was the same as for amplification of *pfhrp2* and *pfhrp3* genes. Parasite strains of *P*. *falciparum* 3D7 and Dd2 were taken as positive and negative controls, respectively, for *pfhrp2* because 3D7 is known to have all *pfhrp2*, *pfhrp3*, and flanking genes while Dd2 is lacking *pfhrp2*and its flanking genes [28]. Another *P*. *falciparum* strain, HB3 isolated from Honduras, was used as a negative control of *pfhrp3* and its flanking genes because of absence of all these genes [29]. These controls were obtained from Malaria Branch at CDC, Atlanta USA.

All PCR products were separated and visualized on a 2% agarose gel. When there was a positive reaction, results was accepted without further repetition. When a negative test result was obtained, the amplification was repeated for confirmation. If the second result was concordant with the first, this was accepted as the final result. However, if the second result was discordant with the previous test result, the experiment was conducted for a third time. The two matching result out of these three were scored as the final result.

#### DNA sequencing of *pfhrp2* and *pfhrp3* gene

All the positive amplification of *pfhrp2* and *pfhrp3* genes (exon2) were sequenced from both directions by using forward and reverse primers of exon2. PCR products were purified by using spin columns (Real Biotech Corporation, Taiwan) according to manufacturer’s instructions and were used in a standard dye terminator (BigDye Terminator v3.1 Cycle Sequencing Kit) DNA sequencing on an Applied Biosystems 3130 XL sequencer.

#### Sequencing result analysis and translation

Sequencing results were analysed by using sequencing analysis software v5.2 (Applied Biosystems) and were assembled using CAP contig assembly programme of BioEdit sequence alignment editor. Nucleotide sequences were translated to amino acid sequences using ExPASy translate tool. Amino acid repeat sequences were identified and given numeric codes [27]. Nucleotide and amino acid sequences were submitted to the NCBI database.

## Results

**Fig 2:**
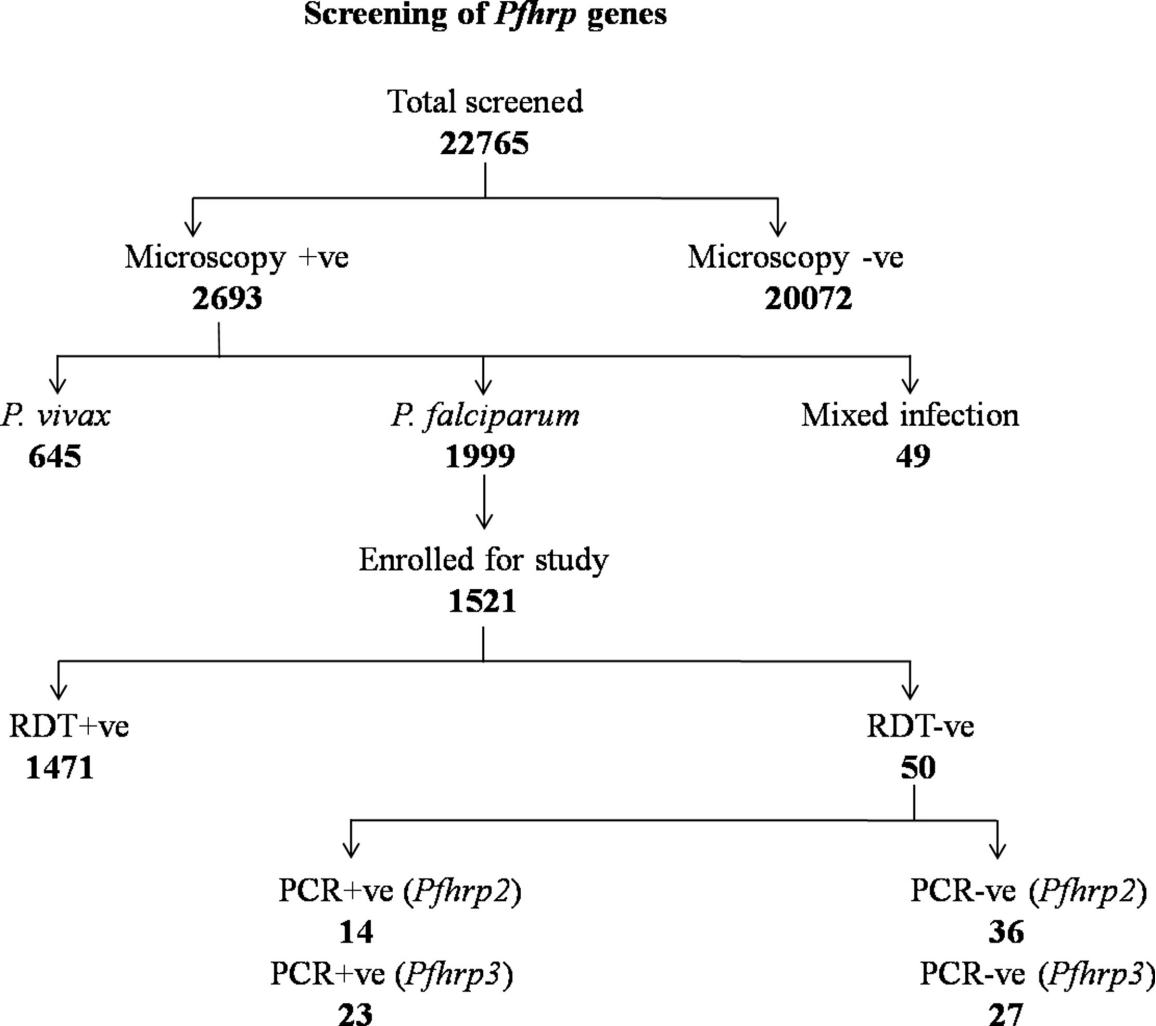
Flow chart showing Screening of malaria cases by microscopy, RDT and polymerase chain reaction (PCR) for *pfhrp2* and *pfhrp3* gene.

From the 16 study sites, a total of 22765 suspected malaria patients were screened for malaria by microscopy of which 2693 were positive for malaria (11.8%). Out of 2693 malaria subjects, 1999 were *P*. *falciparum* (74%), 645 *P*. *vivax* (24%), and 49 had mixed infections of *P*. *falciparum* and *P*. *vivax* (2%) as shown in Fig 2.

### RDT Performance

Out of 1999 *P*. *falciparum* mono-infections, 1521 subjects were enrolled in the study and the rest either left the hospital before enrollment or refused to give consent for enrollment. Bivalent malaria RDT kit was performed among the enrolled patients and overall 3.3% (50/1521) of *P*. *falciparum* confirmed cases were found to be RDT negative (Table 2). Therefore, these 50 RDT negative samples were chosen for further molecular testing to detect if there is lack of *pfhrp2*, *pfhrp3* or their flanking genes. The rest of 1471 specimens were assumed to be carrying a functional *pfhrp2* gene as these specimens showed positive RDT results. The majority of RDT negative cases (2.6%) were from three states (40/1521), i.e. GJ, 11.3% (11/97), OD, 7.1% (19/267), and JH, 4.6% (10/216). The remaining 10 samples were from CG 1.4% (3/214), MH 1.7% (4/234), MP 0.9% (2/226), and TR 0.8% (1/127) while samples from RJ (n = 140) showed 100% matching results between RDT and the microscopy.

**Table 2:** Details of patients screening, positive, enrolled and RDT negative.

State	CHC (Longitude, latitude and mean sea level)	Screened	Pos	Pf	Pf enrolled	RDT negative n (%)	95% CI	HRP2 Negative n (%)	HRP3 Negative n (%)	HRP2 & HRP3 Negative n (%)
Orissa	Bandhugaon, District Koraput	758	349	311	229	18 (7.86)	(4.7–12.1)	12 (5.2)	12 (5.2)	11 (4.8)
	(82.72°E,18.82°N, 870 m)									
	Jagannathpur,District Rayagada	280	40	38	38	1 (2.63)	(0.1–13.8)	0 (0.0)	0 (0.0)	0 (0.0)
	(83.42°E, 19.17°N, 207 m)									
Jharkhand	Jaldega,District Simdega	949	191	146	118	4 (3.39)	(0.9–8.5)	3 (2.5)	2 (1.7)	2 (1.7)
	(84.52°E, 22.62°N, 418m)									
	Bano,District Simdega	2275	272	132	98	6 (6.12)	(2.3–12.9)	2 (2.0)	1 (1.0)	0 (0.0)
	(84.92°E, 22.62°N, 418 m)									
Chhattisgarh	Jagdalpur	4336	386	355	202	0 (0.00)	(0.0–1.8)*	0 (0.0)	0 (0.0)	0 (0.0)
	(82.03°E, 19.07°N, 552 m)									
	Baikunthpur	3116	23	16	12	3 (25.00)	(5.5–57.2)	3 (25.0)	0 (0.0)	0 (0.0)
	(82.55°E, 23.25°N, 556 m)									
Madhya Pradesh	Ranapur,District Jhabua	1503	310	153	125	0 (0.00)	(0.0–2.9) *	0 (0.0)	0 (0.0)	0 (0.0)
	(74.6°E 22.77°N 318 m)									
	Pushprajgarh,District Anuppur	1648	163	142	101	2 (1.98)	(0.2–7.0)	2 (2.0)	2 (2.0)	2 (2.0)
	(81.68°E, 23.1°N,505 m)									
Maharshtra	Malewada,District Gadchiroli	1163	121	116	114	3 (2.63)	(0.5–7.5)	3 (2.6)	3 (2.6)	3 (2.6)
	(80.0°E, 20.10°N, 217 m)									
	Darekasa,District Gondia	1113	135	126	120	1 (0.83)	(0.0–4.6)	0 (0.0)	0 (0.0)	0 (0.0)
	(80.19°E, 21.46°N, 300m)									
Rajasthan	Barabarda,District Pratapgarh	434	5	0	0	0 (0.00)		0 (0.0)	0 (0.0)	0 (0.0)
	(74.8°E, 24.3°N, 580 m)									
	Bekaria,District Udaipur	678	215	158	140	0 (0.00)	(0.0–2.6)*	0 (0.0)	0 (0.0)	0 (0.0)
	(73.68°E, 24.58°N, 600 m)									
Gujarat	Devgadh Baria,District Dahod	2755	318	169	87	11 (12.64)	(6.5–21.5)	10 (11.5)	7 (8.0)	7 (8.0)
	(74°15′E, 22°52′N, 280 m)									
	Lavkar,District Valsad	461	15	10	10	0 (0.00)	(0.0–30.8)*	0 (0.0)	0 (0.0)	0 (0.0)
	(72.93°E, 20.61°N, 13 m)									
Tripura	Manu bazar,South Tripura	168	51	45	45	0 (0.00)	(0.0–7.9)*	0 (0.0)	0 (0.0)	0 (0.0)
	(91°29′E, 23°32′N, 26 m)									
	Santir bazar,South Tripura	1128	99	82	82	1 (1.22)	(0.0–6.6)	1 (1.2)	0 (0.0)	0 (0.0)
	(91°29′E, 23°32′N, 26 m)									
**Total**		**22765**	**2693**	**1999**	**1521**	**50 (3.29)**	**(2.4–4.3)**	**36 (2.4)**	**27 (1.8)**	**25 (1.6)**

* One-sided, 97.5% confidence interval; Pos: Positive for Malaria; Pf: *Plasmodium falciparum*

**Fig 3:**
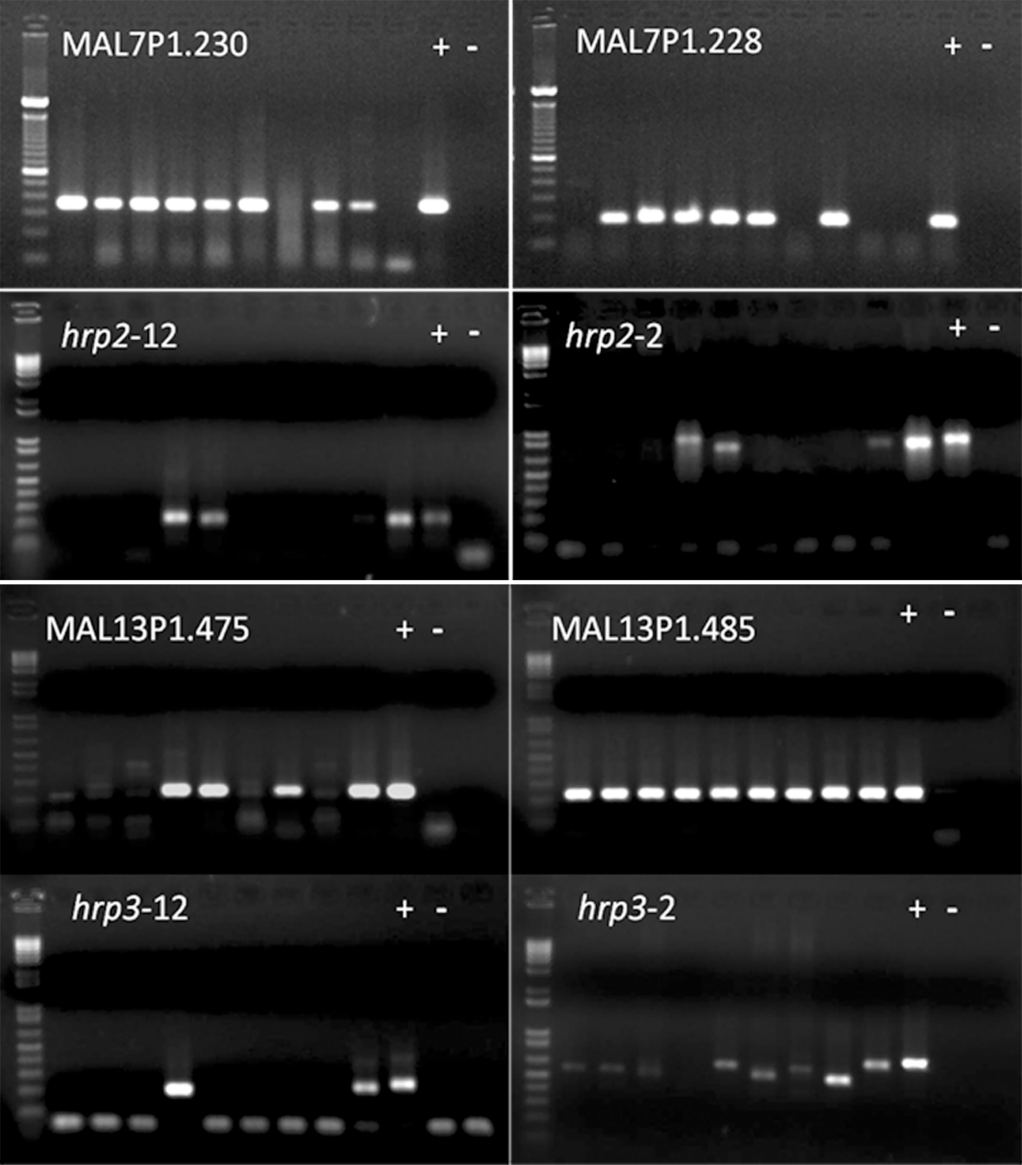
Molecular analysis of RDT negative *P*. *falciparum* samples. Nested PCR amplification of genes; Upstream (MAL7P1.230) and downstream (MAL7P1.228) flanking genes of *pfhrp2*, *pfhrp2* exon 1–2 (*hrp2-12*), *pfhrp2* exon2 (*hrp2-2*), upstream (MAL13P1.475) and downstream (MAL13P1.485) flanking genes of *pfhrp3*, *pfhrp3* exon 1–2 (*hrp3-12*), *pfhrp3* exon2 (*hrp3-2*). Each gel picture shows 100bp marker on 1^st^ well then amplified gene of samples with their positive and negative controls respectively.

A nested PCR reaction that amplifies the 18S rRNA gene of five *Plasmodium* species (*P*. *falciparum*, *P*.*vivax*, *P*. *malariae*, *P*. *ovale*, *and P*. *knowlesi*) infecting humans and two polymorphic *P*. *falciparum* genes *pfmsp1* and *pfmsp2* was undertaken for confirmation of malaria parasite species, and quantity and quality of *P*. *falciparum* parasites DNA (Fig 3). Results of these experiments confirmed that all 50 samples were positive for *P*. *falciparum* (18SrRNA, *pfmsp1* and *pfmsp2* genes), with no evidence of contamination, or co-infection with another parasite species S1 Table. Population marker genes (*pfmsp1 & pfmsp2*) showed 18% (9/50) multiple allelic types among the RDT negative samples. Of these 9 cases, 6 were positive for *pfhrp2* while only 3 cases were negative for *pfhrp2*. The peripheral parasite density was calculated from 1338 cases (46 from RDT negative and 1292 from RDT positive). Out of 46 RDT negative cases 22% (10/46) had parasite density <200/microliter, 30% (14/46) had parasite density between >200–500 /microliter and 48% (22/46) had parasite density >500/microliter. Three of these subjects had very high parasitemia (>5000 parasite/μL, >10,000 parasite/μL, and >20,000 parasite/μL).

### *pfhrp2*, *pfhrp3* and its flanking gene deletions

PCR amplification showed 2.4% samples (36/1521) were lacking the *pfhrp2* gene. Most cases (1.8%) (27/1521) were from the three states i.e. GJ 10.3% (10/97), OD 4.5% (12/267) and JH 2.2% (5/216) (Table 2). The remaining nine samples were from CG 1.4% (3/214), MH 1.3% (3/234), MP 0.9% (2/226), and TR 0.8% (1/127). Only 27 samples showed absence of the *pfhrp3* gene (1.7%) and both *pfhrp2* and *pfhrp3* genes were not found in 1.6% (25/1521) samples by PCR.

Gene deletion patterns of pfhrp2 and pfhrp3 genes

Nineteen different types of *pfhrp2* and *pfhrp3* gene deletion patterns were observed (Table 3). Out of 50 isolates, only seven isolate (14%) had all the *pfhrp2* and *pfhrp3*, and their flanking genes, while 86% isolates had deletion of either *pfhrp2* or *pfhrp3*, or their flanking genes. Further analysis revealed that 14% of isolates lacked all the genes including their flanking genes. Another 14% isolates showed complete lack of *pfhrp2* and its flanking genes while positive for *pfhrp3* and its flanking genes.

**Table 3:** Deletion pattern of pfhrp2 and pfhrp3 and there flanking regions.

PF3D7_0831900 (MAL7P1.230)	PfHRP2 Exon1–2, PF3D7_0831800	PfHRP2 Exon-2, PF3D7_0831800	PF3D7_0831700 (MAL7P1.228)	PF3D7_1372100, (MAL13P1.485)	PfHRP3 Exon1–2, PF3D7_1372200	PfHRP3 Exon -2, PF3D7_1372200	PF3D7_1372400 (MAL13P1.475)	Samples (%)
+	+	+	+	+	+	+	+	7 (14)
+	+	+	+	+	-	-	+	1 (2)
+	+	+	-	+	+	+	+	2 (4)
+	+	-	+	+	+	+	+	1 (1)
+	-	-	+	+	+	+	+	2 (2)
+	-	-	+	+	-	-	+	1 (1)
+	-	-	+	-	-	-	+	2 (2)
+	-	-	+	-	-	-	-	1 (1)
+	-	-	-	-	-	-	+	4 (8)
+	-	-	-	+	+	+	+	1 (2)
-	-	-	-	-	-	-	-	7 (14)
-	-	-	-	-	-	-	+	3 (6)
-	-	-	-	+	-	-	+	1 (2)
-	-	-	+	+	-	-	+	1 (2)
-	+	+	-	+	-	-	+	1 (2)
-	+	+	+	+	+	+	+	3 (6)
-	-	-	+	-	-	-	+	3 (6)
-	-	-	+	-	-	-	-	2 (4)
-	-	-	-	+	+	+	+	7 (14)
							**Total samples**	**50**

#### Variation in *pfhrp2* and *pfhrp3* genes

Exon2 of all 16 positive samples for *pfhrp2* and 23 positive samples for *pfhrp3* were sequenced. A total of 14 different amino acid repeats were identified from *pfhrp2* gene and 8 different amino acid repeat from *pfhrp3* gene (Table 4). Type 1 (AHHAHHVAD), type 2 (AHHAHHAAD), type 7 (AHHAAD), and type 12 (AHHAAAHHEAATH) repeats were observed in 100% of the isolates. Several other repeats occurred only in a few isolates. The sequences were submitted to the Gen Bank database (Gen Bank accession numbers KT 238913-KT 238939).

**Table 4:** Amino acids repeats along with codes observed in *Plasmodium falciparum* histidine rich protein 2 and 3.

Code	Repeat sequences	Antigens observed	
		HRP2	HRP3
1	AHHAHHVAD	+	+
2	AHHAHHAAD	+	-
3	AHHAHHAAY	+	-
4	AHH	+	+
5	AHHAHHASD	+	-
6	AHHATD	+	-
7	AHHAAD	+	+
8	AHHAAY	+	-
9	AAY	+	-
10	AHHAAAHHATD	+	-
11	AHN	-	-
12	AHHAAAHHEAATH	+	-
13	AHHASD	+	-
14	AHHAHHATD	-	-
15	AHHAHHAAN	+	+
16	AHHAAN	-	+
17	AHHDG	-	+
18	AHHDD	-	+
19	AHHAA	+	-
20	SHHDD	-	+
21	AHHAHHATY	-	-
22	AHHAHHAGD	-	-
23	ARHAAD	-	-
24	AHHTHHAAD	-	-

## Discussion

This is first systematic study from India to document the prevalence of the *pfhrp2 and pfhrp3* genes in natural *P*. *falciparum* populations in eight endemic states responsible for 80% of total malaria cases, 85% *Plasmodium falciparum*, and 70% death due to malaria in the country [1]. The study design involved identification of false RDT negative samples by microscopy and PCR amplification of *pfhrp2* and *pfhrp3* genes along with their upstream and downstream flanking genes in order to estimate the extent of deletion around *pfhrp2* and *pfhrp3*. Recent studies of *pfhrp2* gene deletion in natural *P*. *falciparum* population from Peru and other countries [14, 17, 19] have demonstrated the importance of molecular surveillance to detect these deletions as they could lead to false negative diagnoses when *pfhrp2* based RDTs are used. We performed several sets of PCR experiments to confirm that *P*. *falciparum* specimens actually lack these genes. These experiments also ensured that the negative PCR reaction for *pfhrp2* and *pfhrp3* were not due to poor quality or insufficient parasite DNA.

Prior to this study, we did not know whether *P*. *falciparum* parasites with *pfhrp2* deletion were found in the highly endemic states of GJ, OD, and JH. The results of this study confirm that *pfhrp2* deletions occur in natural *P*. *falciparum* parasite populations in India in varying proportions in different highly endemic CHCs (0–25%) and states (0–11%). These results suggest that spontaneous *pfhrp2* deletions occur under natural field conditions with intense transmission as recorded earlier [14, 17, 19]. Moreover, simultaneous infection with different *P*. *falciparum* parasites (multiple genotypes) is also recorded in 18% RDT negative samples. Thus the 36 *pfhrp2* negative out of 50 RDT negative and blood smear positive specimens does not provide the correct estimate of the *pfhrp2* negative frequency of *P*. *falciparum*. This is because one *pfhrp2* positive parasite in a specimen with another *pfhrp2* negative strain may produce a *pfhrp2* positive test results [19].Thus the actual prevalence of *pfhrp2* negative strains could be higher than the estimate reported in this study. In addition, we assumed all 1471 specimens from RDT and microscopy positive individuals were positive for *pfhrp2* and *pfhrp3* genes and therefore not included in the molecular analysis. Given that HRP2 and HRP3 proteins share common epitopes, it is possible that we may have under estimated prevalence of especially *pfhrp3* genes since positive HRP2 RDT test result need not necessarily reveal whether *pfhrp3* is present or not. However, the main goal of this study is to estimate the prevalence of pfhrp2 gene, which codes for HRP2 proteins captured by *P*. *falciparum* specific RDTs.

Although overall results suggested that the majority of persons with *P*. *falciparum* infections are positive using PfHRP2 based RDTs >97% and that PfHRP2 based RDTs are useful for diagnosis especially in regions where skilled microscopists are not available. However, it is of concern that false negative PfHRP2 based RDTs were obtained in some CHCs i.e. Bandhugaon in OD, Devgadh Baria in GJ, and Baikunthpur in CG and thus a negative test results with an RDT based on PfHRP2 (SD Biolines used in this study) does not exclude active infection with *P*. *falciparum*. Moreover, all these 3 CHC's are located in high endemic regions. Multiple infection (MOI) with *P*. *falciparum* strains was limited to 18% and these cases were mainly found in 4 states i.e. OD, JH, GJ and MH.

All samples lacking *pfhrp2* were found in symptomatic patients and occurred at both high and low parasitemia as reported in Peru [16]. This is in contrast to an earlier finding which showed *pfhrp2* gene deletions only in asymptomatic subjects with low parasitemia [19]. It has also been demonstrated that PfHRP3 is likely to compensate for absence of PfHRP2 in diagnosis due to cross-reaction of PfHRP2 with PfHRP3 antibodies [15, 20, 27]. However, in this study 25 subjects, out of 50 lacked both *pfhrp2* and *pfhrp3* genes. Moreover, the role of *pfhrp3* in performance of HRP based diagnostic tests is not well defined [15].

Although among 50 RDT negative samples tested we found higher levels of *pfhrp2* deletion than *pfhrp3*, we cannot generalize this for the whole country without further studies. However, it is worth pointing out that in some countries such as Suriname *pfhrp2* deletion was more common than *pfhrp3* [17]. In contrast, in some countries viz Colombia [14], Peru [15], and Honduras [7] *pfhrp3* deletions are more prevalent than *pfhrp2* deletion.

Three major factors can affect the sensitivity of the PfHRP2 based RDT i.e. parasite density, *pfhrp2* polymorphisms, and *pfhrp2* deletion. The parasite density cannot explain the failure of the detection by PfHRP2 based RDT as there were 48% subjects with high parasitemia showed RDT negative test results for *P*. *falciparum* (>500 parasite/μL). Another factor affecting the sensitivity of the PfHRP2 based RDT is failure of parasite to express the antigen or alteration in PfHRP2 protein sequence due to gene deletion, insertion, pattern of histidine repeats, and SNPs [20, 21, 30]. There were some isolates that were PCR positive for *pfhrp2* gene (14/50) but showed false negative RDT test results. Although we do not know the reason for this false negative test result, it is possible that variation in composition of *pfhrp2* sequence repeat as well as the number of repeat types could have influenced the test results. The amino acid composition of the PfHRP2 protein (organization of the repeats and position of repeats in the antigens) may have an impact on RDT sensitivity [27, 30]. Multiple patterns of deletion (Table 3) within the region of *pfhrp2*, *pfhrp3*, and its flanking genes was present in this study and these kinds of patterns have been reported previously [14]. This kind of multiple gene deletion patterns could be due to their physical location on the chromosomes [7].

The results suggest that the *pfhrp2* and *pfhrp3* deletion phenomenon is also found in India in varying proportions but at a lower proportion. This finding is valuable for laboratories and health policy makers as it showed that PfHRP2 based RDTs can be reliably used in most parts of India where this study was performed. However, even though the prevalence of *pfhrp2/pfhrp3* negative parasites are low in most part of the country, the frequent migration or selection of *pfhrp2* parasites could lead to their spread to other parts of the country as was the case for drug resistance parasites. Moreover, the patients with negative PfHRP2 based RDTs will not be treated for malaria and this could lead to selection of *pfhrp2* negative parasites as NVBDCP policy is to use RDTs for malaria diagnosis and treatment in areas where it is not possible to provide results of microscopy within 24 hrs. If the parasites are undetected by these RDTs, the delay in treatment supports the development of sexual stages and their transmission to mosquitoes during a blood meal. The transmission of parasites undetected by PfHRP2 based RDT would continue, leading to faster selection and dissemination of these genotypes [20]. The frequency of these genotypes would then increase in coming years from these areas like GJ (Devgadh Baria CHC, district Dahod), OD (Bandhugaon CHC, district Koraput), CG (District hospital, district Korea). These districts are also known as highly malarious ones in their respective states.

The strength of this study is that samples were collected from 16 sites in eight endemic states simultaneously during the main transmission season from different geographical regions of the country to understand the pattern of *hrp2* and *hrp3* gene deletions in the areas of different endemicity. Nevertheless, this study has some limitations. The samples were collected only during one season, thus the pattern of the distribution of *pfhrp2* and *pfhrp3* deletions are not known during other seasons of the year. Moreover the prevalence of *pfhrp3* gene deletions estimated in this study may be an underestimate as RDT positive samples were not tested for *pfhrp3* deletion. Another limitation of the study is the number of samples examined from the highly malarious state of Tripura as transmission start much earlier in May as compared to other states where main transmission season starts from July onwards with the onset of rains. A larger and more systematic collection in large parts of Gujarat, Odisha, Jharkhand, and Chhattisgarh will clarify the current prevalence and distribution of *P*. *falciparum* strains with *pfhrp2* and *pfhrp3* deletions in these states. Periodic evaluation of RDT performance and molecular surveillance will be required to ensure the reliable performance of RDTs and to monitor changes in the level of *pfhrp2* deleted parasites in different parts of India.

## Supporting Information

S1 Table PCR amplification of different genes from RDT negative samples. (DOCX)
