# Supplementary material for: Prevalence of pfhrp2 and/or pfhrp3 Gene Deletion in Plasmodium falciparum Population in Eight Highly Endemic States in India
Source: PLoS One. 2016 Aug 12;11(8):e0157949. doi: 10.1371/journal.pone.0157949 (PMC4982695; doi:10.1371/journal.pone.0157949)
Supplement: S1 Table — (DOCX) [file pone.0157949.s001.docx]

**Table S1:** **PCR amplification of different genes from RDT Negative samples.**

| **sample** | **Parasite density/µL** | **18 srRNA** | **MSP1** | **MSP2** | **Up HRP2** | **HRP2 - 12** | **HRP2- 2** | **Down HRP2** | **Up HRP3** | **HRP3 -12** | **HRP3-2** | **Down HRP3** |
| --- | --- | --- | --- | --- | --- | --- | --- | --- | --- | --- | --- | --- |
| 2A049 | 4000.00 | + | + | + | - | - | - | + | - | - | - | + |
| 2A087 | 199.00 | + | + | + | - | - | - | - | - | - | - | + |
| 2A096 | 535.89 | + | + | + | - | + | + | + | + | + | + | + |
| 2A132 | 309.18 | + | + | + | + | + | + | + | + | + | + | + |
| 2A134 | 976.53 | + | + | + | - | + | + | - | + | - | - | + |
| 2A150 | 192.31 | + | + | + | + | + | + | - | + | + | + | + |
| 2A154 | 551.72 | + | + | + | - | - | - | - | - | - | - | + |
| 2A165 | 274.51 | + | + | + | - | - | - | + | + | - | - | + |
| 2A173 | 400.00 | + | + | + | + | + | + | - | + | + | + | + |
| 2A181 | 155.34 | + | + | - | - | - | - | - | + | + | + | + |
| 2A191 | 788.18 | + | + | - | + | - | - | + | + | - | - | + |
| 2A194 | 2115.38 | + | - | + | - | - | - | - | - | - | - | + |
| 2A203 | 621.36 | + | + | + | + | + | + | + | + | + | + | + |
| 2A205 | 384.62 | + | + | + | + | - | - | + | - | - | - | + |
| 2A206 | 497.61 | + | + | - | + | - | - | - | - | - | - | + |
| 2A210 | 541.06 | + | + | + | - | - | - | + | - | - | - | - |
| 2A215 | 150.23 | + | - | + | - | - | - | - | - | - | - | - |
| 2A229 | 157.64 | + | - | + | - | - | - | - | - | - | - | - |
| 2B021 | 2009.30 | + | + | + | + | + | + | + | + | + | + | + |
| 3A023 | 349.51 | + | + | + | - | + | + | + | + | + | + | + |
| 3A025 | 398.01 | + | + | + | - | - | - | + | - | - | - | + |
| 3A031 | 315.27 | + | + | + | - | - | - | - | + | - | - | + |
| 3A071 | 640.00 | + | + | + | - | - | - | - | + | + | + | + |
| 3B002 | 117.65 | + | + | + | + | + | - | + | + | + | + | + |
| 3C006 | 2107.32 | + | + | + | + | + | + | + | + | + | + | + |
| 3C007 | 3407.41 | + | + | + | + | + | + | + | + | - | - | + |
| 3C013 | 1584.16 | + | + | + | + | + | + | + | + | + | + | + |
| 3C048 | 600.00 | + | + | + | - | + | + | + | + | + | + | + |
| 3C049 | 1873.17 | + | + | + | + | - | - | - | + | + | + | + |
| 4b009 | 96.00 | + | + | + | + | - | - | + | + | + | + | + |
| 4B011 | 48.00 | + | + | - | - | - | - | - | + | + | + | + |
| 4B014 | 48.00 | + | + | - | - | - | - | - | + | + | + | + |
| 5B001 | 118.81 | + | + | + | - | - | - | + | - | - | - | - |
| 5B046 | 1773.40 | + | - | + | - | - | - | - | - | - | - | - |
| 6A036 | 7163.64 | + | - | + | - | - | - | - | - | - | - | - |
| 6A051 | 628.10 | + | - | + | - | - | - | - | - | - | - | - |
| 6A062 | 16497.78 | + | - | + | - | - | - | - | - | - | - | - |
| 6B015 | 23960.00 | + | + | + | + | + | + | + | + | + | + | + |
| 8A005 | 233.01 | + | + | + | - | - | - | - | + | + | + | + |
| 8A007 | 533.33 | + | + | + | + | - | - | - | - | - | - | + |
| 8A008 | 238.81 | + | + | + | + | - | - | + | - | - | - | + |
| 8A014 | 234.15 | + | + | + | - | - | - | + | - | - | - | + |
| 8A019 | 313.73 | + | + | + | + | - | - | - | - | - | - | + |
| 8A020 | 316.83 | + | - | + | + | - | - | + | + | + | + | + |
| 8A039 | 360.00 | + | + | + | + | + | + | + | + | + | + | + |
| 8A046 | 1148.15 | + | + | + | + | - | - | - | - | - | - | + |
| 8A073 | 115.38 | + | + | + | - | - | - | - | + | + | + | + |
| 8A074 | 116.50 | + | + | + | - | - | - | - | - | - | - | - |
| 8A080 | 192.31 | + | + | + | + | - | - | + | - | - | - | - |
| 9B015 | 31.31 | + | - | + | - | - | - | - | + | + | + | + |
